# Transferrin-Conjugated Erianin-Loaded Liposomes Suppress the Growth of Liver Cancer by Modulating Oxidative Stress

**DOI:** 10.3389/fonc.2021.727605

**Published:** 2021-08-26

**Authors:** Anhui Yang, Zhen Sun, Rui Liu, Xin Liu, Yue Zhang, Yulin Zhou, Ye Qiu, Xinrui Zhang

**Affiliations:** ^1^School of Life Sciences, Jilin University, Changchun, China; ^2^Department of Pharmacy, Changchun University of Chinese Medicine, Changchun, China

**Keywords:** erianin, transferrin-conjugated liposomes, liver cancer, apoptosis, mitochondrial, oxidative stress

## Abstract

**Background:**

Liver cancer is one of the most malignant human cancers, with few treatments and a poor prognosis. Erianin (ERN) is a natural compound with multiple pharmacological activities that has been reported to have numerous excellent effects against liver cancer in experimental systems. However, its application *in vivo* has been limited due to its poor aqueous solubility and numerous off-target effects. This study aimed to improve the therapeutic efficacy of ERN by developing novel ERN-loaded tumor-targeting nanoparticles.

**Results:**

In this study, ERN was loaded into liposomes by ethanol injection (LP-ERN), and the resulting LP-ERN nanoparticles were treated with transferrin to form Tf-LP-ERN to improve the solubility and enhance the tumor-targeting of ERN. LP-ERN and Tf-LP-ERN nanoparticles had smooth surfaces and a uniform particle size, with particle diameters of 62.60 nm and 88.63 nm, respectively. In HepG2 and SMMC-7721 cells, Tf-LP-ERN induced apoptosis, decreased mitochondrial membrane potentials and increased ERN uptake more effectively than free ERN and LP-ERN. In xenotransplanted mice, Tf-LP-ERN inhibited tumor growth, but had a minimal effect on body weight and organ morphology. In addition, Tf-LP-ERN nanoparticles targeted tumors more effectively than free ERN and LP-ERN nanoparticles, and in tumor tissues Tf-LP-ERN nanoparticles promoted the cleavage PARP-1, caspase-3 and caspase-9, increased the expression levels of Bax, Bad, PUMA, and reduced the expression level of Bcl-2. Moreover, in the spleen of heterotopic tumor model BALB/c mice, ERN, LP-ERN and Tf-LP-ERN nanoparticles increased the expression levels of Nrf2, HO-1, SOD-1 and SOD-2, but reduced the expression levels of P-IKKα+β and P-NF-κB, with Tf-LP-ERN nanoparticles being most effective in this regard. Tf-LP-ERN nanoparticles also regulated the expression levels of TNF-α, IL-10 and CCL11 in serum.

**Conclusion:**

Tf-LP-ERN nanoparticles exhibited excellent anti-liver cancer activity *in vivo* and *in vitro* by inducing cellular apoptosis, exhibiting immunoregulatory actions, and targeting tumor tissues, and did so more effectively than free ERN and LP-ERN nanoparticles. These results suggest that the clinical utility of a Tf-conjugated LP ERN-delivery system for the treatment of liver cancer warrants exploration.

## Introduction

Liver cancer is the world’s third most common cause of cancer-related deaths, due to its frequent recurrence and formation of metastases, and a lack of effective treatments (1, 2). It is most prevalent in Asia and Africa; however, its incidence is increasing in Western countries (3). The primary causes of this globally rising incidence are cirrhosis and chronic hepatitis (4). Currently, radiofrequency ablation, surgery, chemotherapy, radiotherapy, and immunotherapy are the mainstays of liver cancer treatment, but their toxicity and unsatisfactory anti-cancer effects are urgent problems to be solved (5).

One of the key biochemical changes in the development of liver cancer occurs in the network of B-cell lymphoma-2 (Bcl-2) family proteins, which results in the compensatory generation of anti-apoptotic effectors (6). The activation of Bcl-2 associated X protein (Bax) and Bcl-2 homologous antagonist/killer (Bak) protein induces outer mitochondrial membrane permeability and caspase cascade activity (6). Caspase-3 is a crucial pro-apoptotic protein in caspase cascades, and is therefore considered to be a key factor of mitochondrial apoptosis (7). Specifically, caspase-3 amplifies caspase-9 initiation signals *via* the mitochondrial pathway, and also cleaves poly (ADP-ribose) polymerase (PARP), thereby amplifying the apoptotic signal (8).

Oxidative stress triggers the antioxidant response by activating the nuclear factor erythroid 2-related factor 2 (Nrf2) pathway in the liver. Nrf2 then triggers the expression of a variety of downstream cytoprotective genes to maintain cell homeostasis (9). Furthermore, an antioxidant response element in the reverse chain of the Bcl-2 promoter combines with Nrf2 to regulate expression of the *Bcl-2* gene (10). Nrf2 negatively regulates nuclear factor-κB (NF-κB), knockdown of which promotes the transcriptional activity of NF-κB (11). Over-activation of NF-κB, which is normally inactivated by binding to inhibitor of κB alpha (IκB-α). Inhibitor of κB kinase alpha + beta IκB kinase (IKKα+β) releases NF-κB, and the freed NF-κB regulates physiological processes, such as cell proliferation, invasion, and death (12).

Natural products comprise a diverse array of biologically active compounds that have been studied extensively in the field of drug development, especially for cancer therapy (13, 14). For example, erianin (ERN; 2-methoxy-5-[2-(3,4,5-trimethoxyphenyl)-ethyl]-phenol, **Figure S1**) is isolated from *Dendrobium chrysotoxum* Lindl, a widely cultivated species of orchid, and has a variety of pharmacological activities, including anti-cancer activities (15, 16). We recently confirmed that the anti-liver cancer effects of ERN are attributable to its regulation of oxidative stress-mediated mitochondrial apoptosis and the immune response (17). However, ERN has poor aqueous solubility and can only be solubilized in dimethyl sulfoxide, which limits its use *in vivo*.

LPs have similar structures to cell membranes, and are thus well absorbed by cells. Moreover, LPs are easy to modify to improve their drug-loading efficiency, therapeutic utility, and stability (18). Previous studies have successfully used LPs to encapsulate cordycepin, a derivative of adenosine, to improve its solubility and biological activity (19, 20). Transferrin (Tf), an 80-kDa glycoprotein that enables cells to absorb ferric ions (Fe^3+^), is commonly used to actively target therapeutic drug-loaded nanoparticles (such as LPs) to cancer cells, as these cells overexpress Tf receptors on their surfaces (21, 22). In particular, relative to normal cells, liver cells highly overexpress Tf receptors (23).

Based on our previous study (17), in the present study we aimed use *in vivo* and *in vitro* experiments to determine the pro-apoptotic effect of Tf-conjugated LPs loaded with ERN (Tf-LP-ERN) on liver cancer. The resulting data confirmed that Tf-LP-ERN nanoparticles could effectively targeted tumor tissues and cells, and enhanced the immunoregulatory anti-liver cancer effects of ERN.

## Materials and Methods

### Preparation of Liposomes

ERN liposomes (LP-ERN): 1,2-Dioleoyl-3-trimethylammonium-propane (Corden Pharma, Switzerland), egg yolk phosphatidylcholine (Kewpie Corporation, Tokyo, Japan), cholesterol (Nippon Fine Chemical Co., Ltd., Japan), and 1,2-distearoyl-*sn*-glycero-3-phosphoethanolamine-*N*-[methoxy (polyethylene glycol)-2000] (DSPE-mPEG2000) (Lipoid GmbH, Ludwigshafen, Germany) were dissolved in anhydrous ethanol at a molar ratio of 20:45:32:3 to prepare a phospholipid phase. ERN (B20844) (Source Leaf Biological Technology Co., Ltd., Shanghai, China; purity >98.0%) was dissolved in anhydrous ethanol to a concentration of 5 mg/mL. The resulting drug solution and phospholipid phase were thoroughly mixed, and then injected into a magnetically stirred sterile solution of phosphate buffered saline, with the volume ratio of the lipid phase to the water phase=being 1:10). Stirring was continued for 10 min to afford an LP-ERN mixture.

Tf-LP-ERN: The post-insertion method was used to conjugate Tf to LP-ERN (24). A 1:10 molar ratio of a 10 mg/mL Tf (Merck KGaA, St. Louis, MO, USA) solution and a 4 mg/mL solution of Traut’s reagent (2-iminothiolane; Jiamay Biotech Co., Ltd., Beijing, China) were agitated on a shaker for 1 h, and the resulting thiolated Tf was dialyzed. Then, a 1:10 molar ratio of thiolated Tf and Mal-mPEG2000-DSPE (Seebio Biotech Co., Ltd., Shanghai, China) was stirred overnight at room temperature to afford Tf-PEG-DSPE. Finally, a 100:1 molar ratio of LP-ERN and Tf-PEG-DSPE was incubated at 37°C for 30 min to form Tf-LP-ERN. Tf-LP were similarly obtained by co-incubating ERN-free LPs with Tf-DSPE-PEG.

Fluorescent-labeled LPs: A lipid-soluble fluorescent dyes [coumarin 6 (Cou6) (Merck KGaA, St. Louis, MO, USA) or 1,1-dioctadecyl-3,3,3,3-tetramethylindotricarbocyanine iodide (DiR) (Life Technologies, Carlsbad, CA, USA)] was dissolved in anhydrous ethanol, and the resulting solution was dissolved in the lipid phase during the preparation of the above LPs. These two fluorescent dyes can label plasma membranes, and are therefore commonly used to prepare fluorescent labeled LPs.

### Characterization of LP-ERN and Tf-LP-ERN

#### Particle Size-Distribution Analysis

The particle-size distributions of LP-ERN and Tf-LP-ERN nanoparticles were measured at 25°C using a particle size analyzer (Nano ZS90, Malvern Instruments, Malvern, Worcestershire, UK) at 25°C (25).

#### Morphological Investigation

The surface morphologies of LP-ERN and Tf-LP-ERN nanoparticles dropped onto a silicon wafer at 3,000 V were observed by field-emission scanning electron microscopy (FESEM; JSM-6700F, JEOL, Tokyo, Japan) (25).

#### Encapsulation Efficiency Detection

The ERN encapsulation efficiency (EE) of LP-ERN and Tf-LP-ERN nanoparticles was determined using a previously reported procedure, with some modifications (19). Briefly, unencapsulated ERN was separated from the LPs in an ultrafiltration cell (Millipore, USA) equipped with an ultrafiltration membrane with a molecular weight cut-off of 10 kDa, which was centrifuged at 9,000 rpm for 20 min. The concentration of free ERN was recorded, and denoted as F_ERN_. Either LP-ERN or Tf-LP-ERN were lyzed with methanol solution, and the concentrations of ERN released were measured, and denoted as T_ERN_. ERN was quantified on a high-performance liquid chromatography system (E2695, Waters, Milford, MA, USA). The following formula was used to calculate the ERN EE of LP-ERN and Tf-LP-ERN nanoparticles:

$$\text{EE}(\%)=({\text{T}}_{\text{ERN}}-{\text{F}}_{\text{ERN}})/{\text{T}}_{\text{ERN}}\times 100\%$$

### Cell Culture, Cell Viability Assay, and Cellular Uptake Detection

Liver cancer cells [HepG2 (American Type Culture Collection, USA) or SMMC-7721 (China Center for Type Culture Collection, China)] were maintained in Dulbecco’s modified Eagle’s medium supplemented with 10% fetal bovine serum, 1% streptomycin and penicillin, and 0.1% plasmocin prophylactic at 37°C under 5% CO_2_.

HepG2 and SMMC-7721 cells were then seeded into 96-well plates at a concentration of 5,000 cells/well, and subsequently exposed to separate concentration gradients of free ERN, LP-ERN or Tf-LP-ERN for 24 h. 3-(4,5-dimethyl-2-thiazolyl)-2,5-diphenyl-2-*H*-tetrazolium bromide assay, as per our previous study (17).

HepG2 and SMMC-7721 cells were also seeded into 35-mm-diameter glass-bottomed Petri dishes at a density of 100,000 cells/dish, and then exposed to ERN-Cou6, LP-ERN-Cou6 or Tf-LP-ERN-Cou6 nanoparticles (equivalent to 10 nM of ERN and 3 μM of Cou6) for 4 h. Then, a laser-scanning confocal microscope (710 LSMNLO, Carl Zeiss, Jena, Thuringia, Germany) was used to observe the internalization of fluorescent ERN or fluorescent ERN-containing nanoparticles by the liver cancer cells.

### Cell Apoptosis and Mitochondrial Membrane Potential Analyses

HepG2 and SMMC-7721 cells were seeded into six-well plates at a concentration of 3×10^5^ cells/well, and incubated for 24 h. The cells were then exposed to ERN, Tf-LP, LP-ERN or Tf-LP-ERN (to a final ERN concentration of 10 nM) for a further 24 h, and then incubated with 100 μL of Muse™ Annexin V and Dead Cell reagent (Millipore, Billerica, MA, USA) under darkness for 20 min at 25°C. Cell apoptosis was then analyzed using the Muse^®^ Cell Analyzer (EMD Millipore, Billerica, MA, USA).

Another set of six-well plates seeded with HepG2 and SMMC-7721 cells were treated with ERN, Tf-LP, LP-ERN or Tf-LP-ERN (to a final ERN concentration of 10 nM) for 12 h, and then incubated with 10 μM of 5,5′,6,6′-tetrachloro-1,1′,3,3′ tetraethylbenzimidazolylcarbocyanine iodide (JC-1) (Calbiochem, San Diego, CA, USA) for 20 min at 37°C. The changes in the intensity of red and green fluorescence of cells were observed using a fluorescence microscope (Eclipse TE 2000-S, Nikon Corp., Tokyo, Japan).

### Establishment of SMMC-7721-Xenotransplanted Mouse Model

#### SMMC-7721-Xenotransplanted BALB/c Nude Mouse Model

The experimental animal protocol was approved by the Animal Ethics Committee of Jilin University (SY201905019). Twenty-five specific-pathogen-free (SPF) BALB/c nude mice (male, 6 weeks old) (*n* = 5/group) were purchased from Wei-tongli-hua Laboratory Animal Technology Company (Beijing, China), and maintained with adaptive feeding for 1week in barrier facilities under a 12-h light-dark cycle at a temperature of 23 ± 1 °C and a humidity of 50  ± 10%. After this period, a 5 × 10^7^/mL concentration of logarithmic growth-phase SMMC-7721 cells were subcutaneously injected into the right flank of the mice, to generate SMMC-7721-xenotransplanted BALB/c nude mice. When the tumor volumes of these mice reached approximately 100 mm^3^, they were randomly divided into five treatment groups: a control group (tail-vein injected with saline), a Tf-LP group (tail-vein injected with Tf-LP), an ERN group (tail-vein injected with 2 mg/kg of ERN), an LP-ERN group (tail-vein injected with LP-ERN containing 2 mg/kg of ERN), and a Tf-LP-ERN group (tail-vein injected with Tf-LP-ERN containing 2 mg/kg of ERN). These treatments were performed on every second for a period of 14 days. Prior to each treatment, the body weight and tumor size of each mouse were recorded. Tumor volumes were calculated in mm^3^, as length × width × width× 0.5.

After the final treatment, the mice were anesthetized by intraperitoneal injection of 1.5% pentobarbital sodium, photographed, and blood was collected from the caudal vein. The mice were then euthanized, and the tumor tissues were isolated and stored at -80°C, while the organs (the liver, spleen and kidney) were preserved in 4% tissue fixative for subsequent pathological examination.

#### SMMC-7721-Xenotransplanted BALB/c Mice

The experimental animal protocol was approved by the Animal Ethics Committee of Jilin University (SY201905019). Thirty SPF BALB/c mice (male, 8-10 weeks old) (*n* = 6/group) were purchased from Liaoning Changsheng Biotechnology Company (Liaoning, China), adaptively fed for 1 week (as described above), and then intraperitoneally injected with cyclophosphamide (50 mg/kg) on 3 consecutive days. Then, they were seeded with tumors according to the methods in the literature (17). The drug treatment protocol and experimental process were the same as those used for the SMMC-7721-xenotransplanted BALB/c nude mice.

### Evaluation of Distribution of Nanoparticles in SMMC-7721-Xenotransplanted BALB/c Nude Mice

When the tumor volumes of the SMMC-7721-xenotransplanted BALB/c nude mice reached 200 mm^3^, LP-ERN-DiR or Tf-LP-ERN-DiR were injected into mice tail veins to give an ERN concentration of 2 mg/kg. The tissue distributions of LP-ERN-DiR and Tf-LP-ERN-DiR in the mice at 2 h, 4 h and 6 h after injection were observed using a small-animal *in vivo* imaging system (IVIS Kinetic, Caliper, Boston, MA, USA). Finally, the mice were euthanized, tissues were collected, and the fluorescence intensities of tissue types were observed and compared.

### Histopathological Examination

Histopathological examination was performed as in our previous study (19). Slides of liver, spleen, and kidney tissue were stained with hematoxylin and eosin, and then observed using an optical microscope (Nikon Corp., Tokyo, Japan) to detect morphological changes in organ tissue.

### Enzyme-Linked Immunosorbent Assay

The concentrations of tumor necrosis factor-α (TNF-α) (KT2132-A), interleukin-10 (IL-10) (KT2176-A), and chemokine C-C motif ligand 11 (CCL11) (KT30243-A) in blood collected from BALB/c mice were determined using commercial enzyme-linked immunosorbent assay kits (Jiangsu Kete Biotechnology Co., Ltd., Jiangsu, China), according to the manufacturer’s instructions.

### Western Blot Analysis

Tumor tissues obtained from BALB/c nude mice and spleen tissues obtained from BALB/c mice were lyzed and homogenized, and the protein concentrations were measured using a Bicinchoninic Acid Protein Assay Kit (Merck Millipore, Billerica, MA, USA) according to our previous study (17). Protein samples were separated by 10-12% sodium dodecyl sulfate polyacrylamide gel electrophoresis, and then transferred to 0.45 μm polyvinylidene difluoride membranes (Merck Millipore, Burlington, MA, USA). The membranes bearing tumor tissues were blocked with 5% bovine serum albumin solution at 4°C for 6 h, and then exposed to the following primary antibodies at 4°C for 12 h: Bax (ab32503), Bcl-2 antagonist of cell death (Bad) (ab129192), Bcl-2 (ab7973), PARP-1 (ab32138), cleaved PARP-1 (ab32064), cleaved caspase-3 (ab2302), cleaved caspase-9 (ab25758), total caspase-9 (ab25758) (Abcam, Cambridge, MA, USA), total caspase-3 (bs-0081R), p53 upregulated modulator of apoptosis (PUMA) (bs1573R) (Beijing Bioss Biotechnology Co., Ltd., Beijing, China), and glyceraldehyde-3-phosphate dehydrogenase (GAPDH) (E-AB-20032; Elabscience Biotechnology Co., Ltd, Wuhan, China). The membranes bearing spleen tissues were incubated with the following primary antibodies at 4°C for 12 h: Nrf2 (A1244), heme oxygenase-1 (HO-1) (A19062), superoxide dismutase (SOD)-1 (A12537), phospho (P)-NF-κB (AP0475) (Abclonal Biotechnology Co., Ltd., Wuhan, China), SOD-2 (ab13533), P-IKKα+β (ab55341), total (T)-NF-κB (ab7970) (Abcam, Cambridge, MA, USA), T-IKKα+β (bs-10123R) (Beijing Bioss Biotechnology Co., Ltd., Beijing, China), and GAPDH. After washes, the membranes were incubated with goat anti-rabbit (AS014) or goat anti-mouse secondary antibody (AS003) (Abclonal Biotechnology Co., Ltd., Wuhan, China) for 4 h at 4°C. The expression intensity of the proteins was detected using electrochemiluminescence detection kits (Merck Millipore, Billerica, MA, USA) and analyzed with Image J software (NIH, Bethesda, Rockville, MD, USA).

### Statistical Analysis

All values are presented as means ± SDs. Differences were determined by one-way analysis of variance followed by Tukey’s test using SPSS 16.0 software (IBM Corporation, Armonk, NY, USA). A *P*-value less than 0.05 was considered to be a significant difference.

## Results

### Characterization of Tf-LP-ERN Nanoparticles

**Figure 1:**
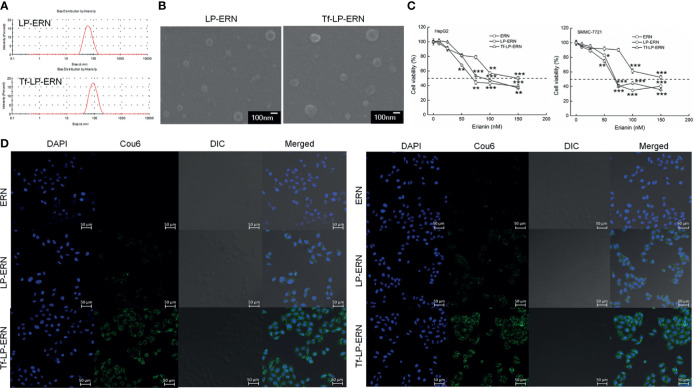
Characterization of LP-ERN and Tf-LP-ERN. **(A)** The particle size distribution of LP-ERN and Tf-LP-ERN were uniform, both less than 100 nm (n = 3). **(B)** Field emission scanning electron microscopy (FESEM) images of LP-ERN and Tf-LP-ERN indicating their uniformly spherical (30,000 ×) (Scale bar: 100 nm) (n = 3). **(C)** ERN, LP-ERN and Tf-LP-ERN reduced the viability of HepG2 and SMMC-7721 cells in a concentration gradient (n = 6). **(D)** ERN, LP-ERN and Tf-LP-ERN can be taken up by HepG2 and SMMC-7721 cells (magnification: 200, scale bar: 50 μm) (n = 3). Liposomes were labelled with Coumarin 6 (Cou6) and the nuclei were labelled with 4’,6-diamidino-2-phenylindole (DAPI). *P < 0.05, **P < 0.01 and ***P < 0.001 *vs*. control cells.

The particle size of the LP-ERN was 62.60 nm and their polydispersity index (PDI) was 0.137, whereas these parameters for the Tf-LP-ERN was 88.63 nm and 0.165, respectively (**Figure 1A** and **Table 1**). The LP-ERN and Tf-LP-ERN nanoparticles had smooth surfaces and uniform particle sizes, as shown by the FESEM results (**Figures 1A, B**). There was no significant change in the PDI of the nanoparticles, indicating that the LPs prepared by ethanol injection had good reproducibility and a uniform particle-size distribution. The EE values of the LP-ERN and Tf-LP-ERN nanoparticles were 69.5% and 68.5%, respectively (**Table 1**). The EE of the LPs modified with Tf was almost unchanged, indicating that the phospholipids effectively encapsulated ERN.

**Table 1:** Characterization of LP-ERN and Tf-LP-ERN properties.

Sample	Particle size (nm)	PDI	EE(%)
LP-ERN	62.60 ± 3.11	0.137 ± 0.05	69.5 ± 0.8
Tf-LP-ERN	88.63 ± 4.21	0.165 ± 0.03	68.5 ± 1.3

The data were analyzed using a one-way analysis of variance and expressed as mean ± S.D. (n = 3).

### Tf-LP-ERN Induced Mitochondrial Apoptosis in Liver Cancer Cells

In HepG2 cells, the IC_50_ values of the ERN, LP-ERN, and Tf-LP-ERN were 136 nM, 99.48 nM, and 100.82 nM, respectively, whereas their IC_50_ values in SMMC-7721 cells were 147 nM, 100.23 nM, and 109.12 nM, respectively (**Figure 1C**). The Tf-modified ERN-containing nanoparticles were absorbed more effectively by liver cancer cells than the non-Tf-modified ERN-containing nanoparticles, as shown by the increased green fluorescence of the Tf-LP-ERN nanoparticle-treated cells (**Figure 1D**).

**Figure 2:**
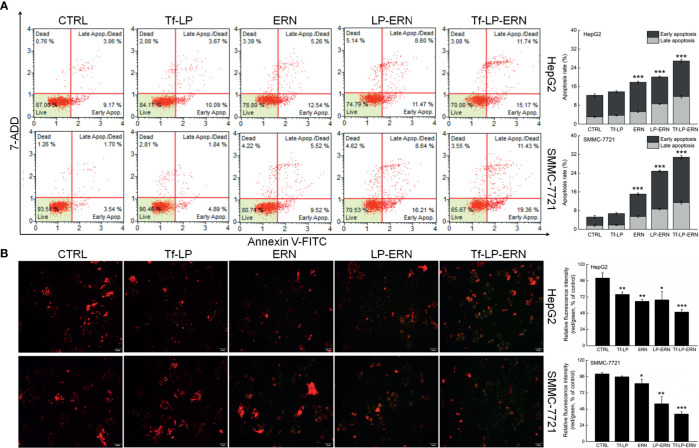
ERN, LP-ERN and Tf-LP-ERN induced apoptosis of HepG2 and SMMC-7721 cells by reducing the mitochondrial membrane potential, among which, Tf-LP- ERN showed the best efficacy. ERN, LP-ERN and Tf-LP-ERN strongly **(A)** enhanced the apoptosis rate (n = 3) and **(B)** reduced the liver cancer cells mitochondrial membrane potential (magnification: 200, scale bar: 50 μm) (n = 3). Qualitative data of mitochondrial membrane potential are expressed as the ratio of red to green fluorescence intensity. *P < 0.05, **P < 0.01 and ***P < 0.001 *vs.* control cells.

Exposure of liver cancer cells to ERN, LP-ERN and Tf-LP-ERN led to different extents of cell apoptosis: the Tf-LP-ERN caused 26.91% and 30.79% early/late apoptosis in HepG2 and SMMC-7721 cells, respectively (**Figure 2A**). Decreases in the MMP are the first indication of apoptosis (26), and treatment with ERN, LP-ERN or Tf-LP-ERN all strongly reduced the MMP in liver cancer cells. The greatest reduction was generated by Tf-LP-ERN nanoparticle treatment, as they accumulated to the greatest extent in cells, which was demonstrated by the Tf-LP-ERN nanoparticle-treated cells having the highest intensity of green fluorescence (**Figure 2B**).

Tf-LP nanoparticle treatment failed to influence liver cancer cells’ apoptosis rate, and the MMPs in these cells suggested that the liposomal material was non-cytotoxic (**Figure 2**).

### Tf-LP-ERN Inhibited Xenografted Tumor Growth in Nude Mice

**Figure 3:**
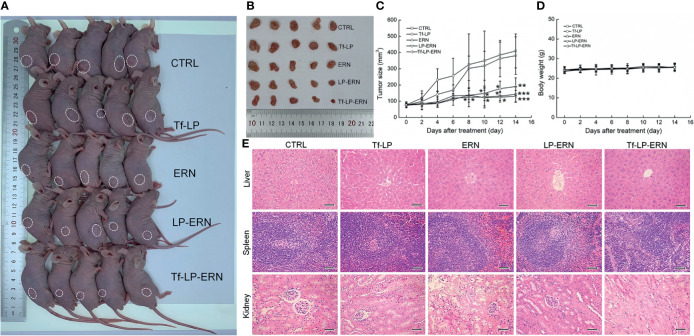
ERN, LP-ERN and Tf-LP-ERN suppressed the tumor growth and showed biological safety in SMMC-7721-xenotransplanted BALB/c nude mice. The tumor volume comparison in **(A)** SMMC-7721-xenotransplanted BALB/c nude mice, and **(B)** their collected tumors. **(C)** Tf-LP-ERN significantly reduced the tumor volume of SMMC-7721-xenotransplanted BALB/c nude mice after 14-d administration (n = 5). Tf-LP, ERN, LP-ERN and Tf-LP-ERN showed no significant effects **(D)** on the body weight and **(E)** histopathologic changes including liver, spleen and kidney in SMMC-7721-xenotransplanted mice (magnification: 200, scale bar: 50 μm). *P < 0.05, **P < 0.01 and ***P < 0.001 *vs.* CTRL mice.

Compared with control treatment mice, two weeks of administration of ERN, LP-ERN or Tf-LP-ERN significantly inhibited the growth of tumors in SMMC-7721-xenotransplanted BALB/c nude mice (*P* < 0.05, **Figures 3A–C**). The Tf-LP-ERN showed the best tumor volume-suppressive effects, due to the enhanced permeability and retention (EPR) effect of LPs; however, Tf-LP did not suppress tumor growth (**Figures 3A–C**).

Neither ERN nor nanoparticles (Tf-LP, LP-ERN, or Tf-LP-ERN) had significant effects on the body weight or organ morphology of SMMC-7721-xenotransplanted BALB/c nude mice (**Figures 3D, E**).

### Tf-LP-ERN Targeted Tumor Tissues to Induce Mitochondrial Apoptosis

**Figure 4:**
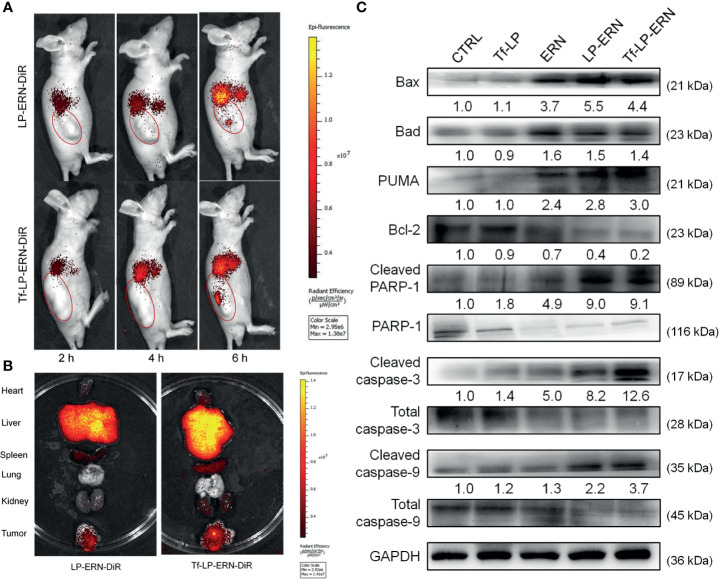
Tf-LP-ERN alleviated cancer development by targeting tumor tissues and caused mitochondrial apoptosis. **(A)** LP-ERN and Tf-LP-ERN labelled by DiR gradually accumulate to tumor tissues in SMMC-7721-xenotransplanted BALB/c nude mice (n = 3/group). **(B)** The tissue distribution of LP-ERN and Tf-LP-ERN in heart, liver, spleen, lung, kidney and tumor after 6 h of tail vein injection in SMMC-7721-xenotransplanted BALB/c nude mice (n = 3/group). **(C)** ERN, LP-ERN and Tf-LP-ERN regulated the expressions of Bcl-2 family members and caspases in tumor tissues of SMMC-7721-xenotransplanted BALB/c nude mice. Quantification data were normalized by GAPDH or the corresponding total proteins and were reported as the folds of those from the corresponding CTRL mice (n = 3).

LP-ERN-DiR and Tf-LP-ERN-DiR nanoparticles were injected intravenously into SMMC-7721-xenotransplanted BALB/c nude mice to investigate their tumor-targeting abilities. Six hours after administration, the fluorescence intensity at the tumor site in the Tf-LP-ERN-DiR nanoparticle treatment group was higher than that in the LP-ERN-DiR nanoparticle treatment group, which indicated that Tf enhanced the tumor-targeting ability of the nanoparticle drug-delivery system (**Figure 4A**).

Tissues were collected 6 h after treatment to further examine the distribution of fluorescence. The liver and spleen tissue exhibited high fluorescence, due their roles in metabolism and reticuloendothelial processing (**Figure 4B**).

In the tumor tissues of SMMC-7721-xenotransplanted BALB/c nude mice, Tf-LP-ERN strongly enhanced the expression levels of Bax, Bad, PUMA, cleaved RARP-1, cleaved caspase-3 and caspase-9, and decreased the expression level of Bcl-2 (**Figure 4C**). This suggests that treatment with Tf-LP-ERN may alleviate the development of liver cancer by inducing mitochondrial apoptosis.

### Tf-LP-ERN Inhibited Xenografted Tumor Growth in BALB/c Mice by Enhancing Immune Function

**Figure 5:**
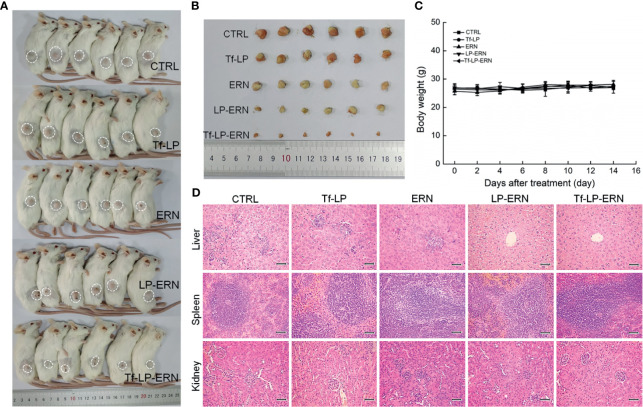
ERN, LP-ERN and Tf-LP-ERN suppressed the tumor growth and showed biological safety in SMMC-7721-xenotransplanted BALB/c mice. The tumor volume comparison in **(A)** SMMC-7721-xenotransplanted BALB/c mice, and **(B)** their collected tumors (n = 6). Tf-LP, ERN, LP-ERN and Tf-LP-ERN showed no significant effects **(C)** on the body weight and **(D)** histopathologic changes including liver, spleen and kidney in SMMC-7721-xenotransplanted mice (magnification: 200, scale bar: 50 μm).

Treatment of SMMC-7721-xenotransplanted BALB/c mice with ERN, LP-ERN nanoparticles, or Tf-LP-ERN nanoparticles inhibited tumor growth without affecting body weight or organ morphology (**Figure 5**). Tf-LP-ERN nanoparticle treatment had the greatest suppressive effects on tumor growth, whereas Tf-LP nanoparticle treatment did not suppress tumor growth (**Figures 5A, B**).

**Figure 6:**
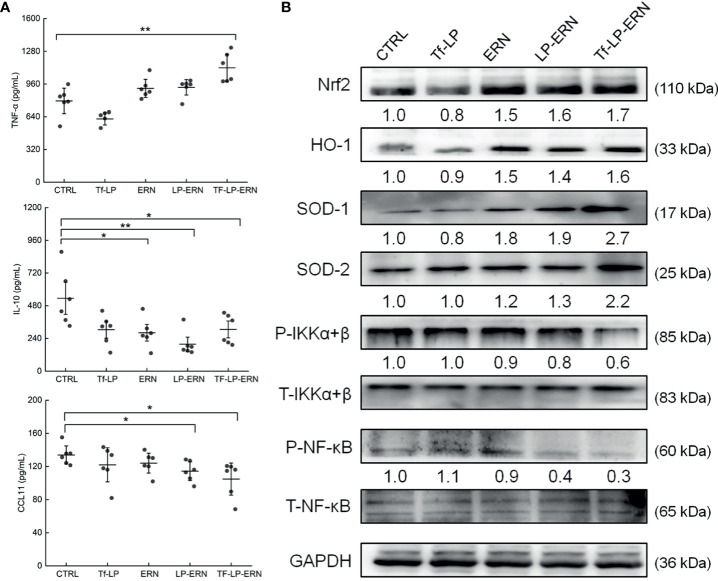
The effects of ERN, LP-ERN and Tf-LP-ERN on inflammatory factor in serum of SMMC-7721-tumor-bearing mice. **(A)** ERN, LP-ERN and Tf-LP-ERN significantly reduced the levels of IL-10, CCL11, and enhanced the level of TNF-α (n = 6). **(B)** In spleens of SMMC-7721-xenografted mice, ERN, LP-ERN and Tf-LP-ERN increased the expression levels of Nrf2 and its downstream proteins HO-1, SOD-1, SOD-2, while reduced the expression levels of P-IKKα+β, and P-NF-κB, among which, Tf-LP-ERN showed the best efficacy. Quantification data were normalized by GAPDH or the corresponding total proteins and were reported as the folds of those from the corresponding CTRL mice (n = 3). The data were analyzed using a one-way analysis of variance and expressed as means ± S.D. (n = 3). *P < 0.05 and **P < 0.01 *vs*. control cells.

In SMMC-7721-tumor-bearing mice, Tf-LP-ERN treatment increased the serum concentration of TNF-α (*P* < 0.01) and decreased the serum concentrations of IL-10 (*P* < 0.05) and CCL11 (*P* < 0.05) (**Figure 6A**). Nrf2 is a key transcription factor that regulates the expression of cytoprotective genes in various types of cells or tissues, which is crucial for defending cells against oxidative stress (27). The spleens of SMMC-7721-xenografted mice treated with ERN, LP-ERN or Tf-LP-ERN exhibited increased levels of expression of Nrf2 and its downstream proteins HO-1, SOD-1, SOD-2, and reduced the levels of expression of P-IKKα+β, and P-NF-κB. Tf-LP-ERN nanoparticle treatment had the greatest effects in this regard (**Figure 6B**).

## Discussion

Liver cancer is mainly caused by inflammation, such as that generated by hepatitis B and C viruses (28). Our previous study found that ERN exerts anti-liver cancer effects by regulating mitochondrial apoptosis and the immune response (17); however, its biopharmaceutical applications have been hampered due to its poor aqueous solubility and tumor-targeting ability. Therefore, in this study we aimed to develop a novel nanoparticle drug-delivery system, Tf-LP-ERN, to enhance the aqueous solubility and tumor-targeting ability of ERN. Our investigations confirmed that Tf-LP-ERN nanoparticles effectively targeted tumor tissues and exhibited increased anti-liver cancer efficacy, as they enabled ERN to strongly affect immunoregulatory pathways in liver cancer cells and in a xenografted tumor mouse model.

The penetration of drugs or drug carriers into tumor tissues, and their accumulation in such tissues, is affected by their particle size; as such, the particle size of LPs is their most practical feature (29). Drug-loaded LPs with a particle size of 60-200 nm selectively penetrate tumor blood vessels, and thereby accumulate in tumor tissues rather than normal tissues, which greatly reduces the adverse effects of drugs on normal tissues (30). The PDI is used to measure the degree of uniformity of particle sizes in a sample (31). The PDI value of Tf-LP-ERN nanoparticles, which had a particle size of 88.63 nm, was approximately 0.165, indicating that the LPs prepared by the ethanol injection method had high uniformity and stability. This accounted for their selective accumulation in tumor tissues.

A significant step in cell apoptosis is the destruction of the MMP, which largely occurs in the early stages of apoptosis. LP-ERN or Tf-LP-ERN nanoparticle treatment of HepG2 and SMMC-7721 cells suppressed cell viability, enhanced cellular apoptosis rate, reduced cell MMPs, and increased cell uptake of ERN, which are all suggestive of anti-tumor activity. Furthermore, such treatment increased the accumulation of ERN in tumor tissues, and thus LP-ERN or Tf-LP-ERN nanoparticle treatment effectively inhibited tumor growth in SMMC-7721-xenografted nude mice. Notably, this was achieved without concomitant effects on the body weight or organs structure of mice, which confirmed the safety of these treatments. Tissue distribution data confirmed these observations, and revealed that Tf-LP-ERN nanoparticles accumulated more in tumor tissues over time than LP-ERN nanoparticles or free ERN.

The Tf receptor has a special extracellular structure that mediates the endocytosis of Tf, enabling the cellular absorption of its Fe^3+^ cargo, and this receptor is overexpressed on the surface of tumor cells relative to normal cells (23). This is because rapidly proliferating tumor cells require high concentrations of Fe^3+^, and accounts for the tumor-targeting ability of Tf (32). Therefore, Tf-conjugated LPs are capable of tumor targeting. For example, Tf modified paclitaxel-loaded LPs were shown to have greater tumor-inhibitory activity than paclitaxel itself (33), and the use of a Tf-LP system to deliver 1,2-Dihydroquinoline 2 increased cell uptake by approximately 3.7 times (34). Tf-conjugated LPs have negatively charged surfaces, which decreases electrostatic interactions between Tf-conjugated LPs, between Tf-conjugated LPs and cell membranes and serum proteins, thereby prolonging the presence of Tf-conjugated LPs in blood circulation and reducing their non-specific uptake by ordinary cells (35). Our results confirmed that Tf receptor-mediated endocytosis on the surface of tumor cells increased the uptake of ERN.

When an apoptotic stimulus occurs, Bad heterodimerizes with B-cell lymphoma-extra large (Bcl-xL), which releases Bax from Bcl-xL. Bax then translocates to the mitochondria and inserts its N-terminus into the outer mitochondrial membrane, which induces the mitochondrial membrane to become permeable (36, 37). Bcl-2 inhibits this process by interacting with Bax (38). The MMP is depolarized by Bax translocation and Bcl-2 dissipation, and then caspase-9 and its downstream counterpart, caspase-3, are activated, which leads to the initiation of apoptosis (39, 40). PUMA, a BH3-only protein and a pro-apoptotic member of the Bcl-2 family, indirectly inhibits anti-apoptotic Bcl-2 proteins such as Bax by inducing mitochondrial dysfunction and caspase activation (41). ERN, LP-ERN and Tf-LP-ERN enhanced the expression levels of Bax, Bad, and PUMA, promoted the cleaved caspase-3, cleaved-9 and PARP-1, and reduced the expression level of Bcl-2, which induced apoptosis and thus to inhibited tumor growth in xenotransplanted BALB/c nude mice. Tf-LP-ERN had the greatest pro-apoptotic effect, as they more effectively targeted tumors than the other treatments.

We previously confirmed that ERN inhibits the growth of liver cancer tumors due to its immunoregulatory effects (17). The spleen is the body’s largest immune organ and the source of many immune cells (42). The immune response in the spleen is closely related to the development of liver cancer, which is accompanied by oxidative stress. Tf-LP-ERN improved the anti-tumor effect of ERN without affecting its apparent mechanism of action. Moreover, Tf-LP-ERN not only accumulated in tumors and the liver but also in the spleen, which explains why splenic protein concentrations in Tf-LP-ERN-treated mice were better regulated than those in ERN-treated mice. Tf-LP-ERN also enhanced the expression levels of Nrf2 and its downstream proteins, which were responsible for the suppression of the phosphorylated activation of NF-κB. HO-1 and SOD eliminate free radicals, as part of the body’s defense against diseases (43, 44). Nrf2 and NF-κB are mutually regulated. Accordingly, the downregulation of Nrf2 can increase the phosphorylation of IκB-α, resulting in the phosphorylation of NF-κB (45). TNF-α is a pleiotropic cytokine that plays an important role in the development and progression of tumors IL-10 is an anti-inflammatory and immunosuppressive cytokine that inhibits the activity of macrophages and the secretion of inflammatory cytokines, such as IL-6 and TNF-α. IL-10 therefore plays an critical role in the negative feedback regulation of the immune response and the inflammatory response (47, 48). In addition, IL-10 enables tumor cells to evade host immune-system defenses, and promotes their metastasis (49). In many inflammatory responses, NF-κB signaling is involved in the secretion of the chemokine CCL11, in coordination with other signaling mechanisms (50). CCL11 inhibits the differentiation of dendritic cells and enhances the polarization of T-helper 2 cells (51, 52). In addition, CCL11 promotes angiogenesis, and its overexpression is closely related to the occurrence and progression of cancer (53). Thus, enhancement of the body’s immune response adversely affects the tumor microenvironment, which induces endogenous tumor-cell apoptosis (54).

We acknowledge that this study has some limitations. Further experiments are needed to complete the evaluation of the drug delivery system, and further investigation is needed to show how ERN interacts with the tumor microenvironment.

## Conclusion

LP-ERN nanoparticles improved the solubility of ERN, and Tf-LP-ERN nanoparticles more effectively targeted tumor cells than LP-ERN nanoparticles, leading to better anti-liver cancer activity *in vivo.* Consistent with previous studies of ERN, we found that the enhanced anti-liver cancer effects of Tf-LP-ERN were due to immunoregulation.

Our novel Tf-conjugated nanoparticle-based ERN-delivery system was highly efficient, accurately targeted tumor cells, and had a good safety profile in a mouse model. This suggests that it warrants further exploration as a potential treatment for liver cancer.

## Data Availability Statement

The original contributions presented in the study are included in the article/**Supplementary Material**. Further inquiries can be directed to the corresponding authors.

## Ethics Statement

The animal study was reviewed and approved by the Animal Ethics Committee of Jilin University.

## Author Contributions

YQ and XZ designed the experiments, draft and revised the manuscript. AY, ZS, RL, and XL performed the experiments and analyzed the data. YZha and YZho analyzed the data. All authors contributed to the article and approved the submitted version.

## Funding

This study is supported by the Science and Technology Develop Project in Jilin Province of China under the grant (No. 20200201122JC and 20200201030JC) and the Science and Technology Research Project, Education Department of Jilin Province of China under the grant No. JJKH20200322KJ.

## Conflict of Interest

The authors declare that the research was conducted in the absence of any commercial or financial relationships that could be construed as a potential conflict of interest.

## Publisher’s Note

All claims expressed in this article are solely those of the authors and do not necessarily represent those of their affiliated organizations, or those of the publisher, the editors and the reviewers. Any product that may be evaluated in this article, or claim that may be made by its manufacturer, is not guaranteed or endorsed by the publisher.

## Abbreviations

Bad, Bcl-2 antagonist of cell death; Bak, Bcl-2 homologous antagonist/killer; Bax, Bcl-2 associated X protein; Bcl-2, B-cell lymphoma-2; Bcl-xL, B-cell lymphoma-extra large; CCL11, chemokine C-C motif ligand 11; Cou6, Coumarin 6; DiR, 1,1-dioctadecyl-3,3,3,3-tetramethylindotricarbocyanine iodide; EE, encapsulation efficiency; EPR, enhanced permeability and retention; ERN, Erianin; FESEM, field emission scanning electron microscopy; GAPDH, glyceraldehyde-3-phosphate dehydrogenase; HO-1, heme oxygenase-1; IκB-α, inhibitor of κB alpha; IKKα+β, inhibitor of κB kinase alpha + beta; IL-10, interleukin-10; LP-ERN, erianin liposomes; MMP, mitochondrial membrane potential; NF-κB, nuclear factor-κB; Nrf2, nuclear factor erythroid 2-related factor 2; P-, phospho-; PARP, poly (ADP-ribose) polymerase; PDI, polydispersity index; PUMA, p53 upregulated modulator of apoptosis; SOD, superoxide dismutase; SPF, specific-pathogen-free; T-, total-; Tf, transferrin; Tf-LP-ERN, Tf-conjugated liposomes loaded with ERN; TNF-α, tumor necrosis factor-α.
