# Supplementary material for: Transferrin-Conjugated Erianin-Loaded Liposomes Suppress the Growth of Liver Cancer by Modulating Oxidative Stress
Source: Front Oncol. 2021 Aug 26;11:727605. doi: 10.3389/fonc.2021.727605 (PMC8427311; doi:10.3389/fonc.2021.727605)
Supplement: Supplementary file 1 [file DataSheet_1.docx]

Supplementary Material

## Supplementary Figures





**Supplementary Figure 1.** Structural formula of Erianin.
